# SSCC: A Novel Computational Framework for Rapid and Accurate Clustering Large-scale Single Cell RNA-seq Data

**DOI:** 10.1016/j.gpb.2018.10.003

**Published:** 2019-06-13

**Authors:** Xianwen Ren, Liangtao Zheng, Zemin Zhang

**Affiliations:** BIOPIC, Beijing Advanced Innovation Center for Genomics, and School of Life Sciences, Peking University, Beijing 100871, China

**Keywords:** Single cell, RNA-seq, Clustering, Subsampling, Classification

## Abstract

**Clustering** is a prevalent analytical means to analyze **single cell** RNA sequencing (scRNA-seq) data but the rapidly expanding data volume can make this process computationally challenging. New methods for both accurate and efficient clustering are of pressing need. Here we proposed Spearman **subsampling**-clustering-**classification** (SSCC), a new clustering framework based on random projection and feature construction, for large-scale scRNA-seq data. SSCC greatly improves clustering accuracy, robustness, and computational efficacy for various state-of-the-art algorithms benchmarked on multiple real datasets. On a dataset with 68,578 human blood cells, SSCC achieved 20% improvement for clustering accuracy and 50-fold acceleration, but only consumed 66% memory usage, compared to the widelyused software package SC3. Compared to *k*-means, the accuracy improvement of SSCC can reach 3-fold. An R implementation of SSCC is available at https://github.com/Japrin/sscClust.

## Introduction

Single cell RNA sequencing (scRNA-seq) has revolutionized transcriptomic studies by revealing the heterogeneity of individual cells with high resolution [1], [2], [3], [4], [5], [6]. Clustering has become a routine analytical means to identify cell types, depict their functional states, and infer potential cellular dynamics [4], [5], [6], [7], [8], [9], [10]. Multiple clustering algorithms have been developed, including Seurat [11], SC3 [12], SIMLR [13], ZIFA [14], CIDR [15], SNN-Cliq [16], and Corr [17]. These algorithms improve the clustering accuracy of scRNA-seq data greatly but often have high computational complexity, impeding the extension of these elegant algorithms to large-scale scRNA-seq datasets. With the rapid development of scRNA-seq technologies, the throughput has increased from initially hundreds of cells to tens of thousands of cells in one run nowadays [18]. Integrative analyses of scRNA-seq datasets from multiple runs or even across multiple studies further exacerbate the computational difficulties. Thus, algorithms that can cluster single cells both efficiently and accurately are needed.

To handle multiple large-scale scRNA-seq datasets, *ad hoc* computational strategies have been proposed by downsampling or convoluting large datasets to small ones [12], [19], [20], [21] or by accelerating the computation with new software implementation [22]. Such strategies have reached variable levels of success but have not adequately addressed the challenges. Considering the importance of efficient and accurate clustering tools for analyses of large-scale scRNA-seq data, here we propose a new computational framework, the Spearman subsampling-clustering-classification (SSCC), based on machine learning techniques, including feature engineering and random projection, to achieve both improved clustering accuracy and efficacy. Benchmarking on various scRNA-seq datasets demonstrates that compared to the current solutions, SSCC can reduce the computational complexity from O(n^2^) to O(n) while maintaining high clustering accuracy. Moreover, flexibility of the new computational framework allows our methods to be further extended and adapted to a wide range of applications for scRNA-seq data analysis.

## Method

### Framework overview

**Figure 1:**
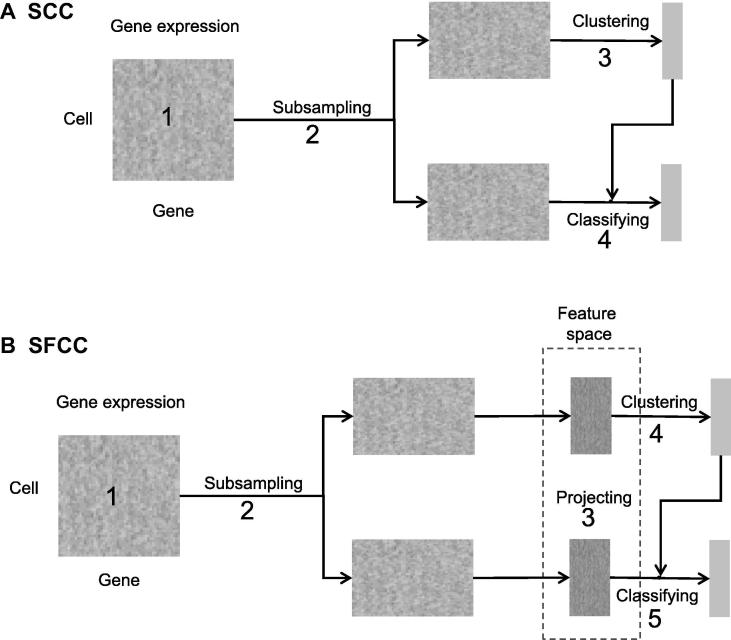
**Two computational frameworks for rapid clustering large-scale scRNA-seq datasets** **A.** The original computational framework proposed in SC3 (referred to SCC) consists of four main steps: (1) constructing the gene expression matrix; (2) dividing the matrix into two parts through cell subsampling; (3) clustering the subsampled cells; and (4) classifying the unsampled cells into clusters. **B.** The new computational framework proposed in this study (referred to SFCC). A feature construction step is added before clustering and classification. The whole framework comprises five steps: (1) constructing the gene expression matrix; (2) dividing the matrix into two parts through cell subsampling; (3) projecting the subsampled/unsampled cells into feature space; (4) clustering the subsampled cells in the feature space; (5) classifying the unsampled cells into clusters in the feature space. scRNA-seq, single cell RNA-sequencing; SC3, single-cell consensus clustering; SCC, subsampling-clustering-classification; SFCC, subsampling-featuring-clustering-classification.

Among the available solutions to handle large scRNA-seq datasets, clustering with subsampling and classification [12], [19] has linear complexity, *i.e.*, O(n). Such a framework generally consists of four steps (Figure 1A). (1) a gene expression matrix is constructed by data preprocessing techniques including gene and cell filtration and normalization; (2) cells are divided into two subsets for clustering and classification separately by subsampling; (3) the subsetted cells for clustering are grouped into clusters using *k*-means [23], hierarchical clustering [24], density clustering [25], or algorithms developed specially for scRNA-seq; and (4) supervised algorithms such as *k*-nearest neighbors [26], support vector machines (SVMs) [27], or random forests [28] are used to predict the labels of other cells based on the clustering results at the third step. For simplicity, we referred this existing framework as subsampling-clustering-classification (SCC). Because clustering is time-consuming and memory-exhaustive, limiting this step to a small subset of cells through subsampling can greatly reduce the computational cost from O(n^2^) to O(n) by leveraging the efficiency of supervised machine learning algorithms. However, classifiers built on the original gene expression data of a small subset of cells may be flawed and biased due to noise of the raw data and small number of cells, thus impairing the accuracy of label assignment for the total cells.

Here we proposed a new computational framework for clustering large scRNA-seq data by adding a feature engineering/projecting step into SCC (Figure 1B). Similar to SCC, a gene expression matrix is first constructed through gene and cell filtrations and normalization (Step 1, Figure 1B), and is then split randomly into two subsets for clustering and classification separately (Step 2; Figure 1B). Unlike SCC, which directly uses the raw data of gene expression, our new framework projects cells into a feature space (Step 3; Figure 1B) for clustering (Step 4; Figure 1B) and classification (Step 5; Figure 1B). As the new framework is characterized by a subsampling-featuring-clustering-classification strategy, we named it as SFCC. Specifically, we divide feature construction into two steps: (1) feature extraction techniques are applied to cells subject to clustering; and (2) according to the selection of feature extraction methods, cells for classification are then projected into the built feature space. Many established techniques in the machine learning field can be exploited in these two steps. For example, principal component analysis (PCA) [29] can be used to first construct features for cells undergoing clustering while the resultant loading vectors can be used as linear transformations to project cells for classification into the feature space. Selecting different algorithms in each step of the SFCC framework would then form different pipelines for clustering large-scale scRNA-seq datasets. To reduce the total number of algorithmic combinations, here we focus on comparing the performance between various feature engineering algorithms. We hold algorithms for gene and cell filtration, normalization, subsampling, and classification as the algorithms frequently used in practice. The existing SCC strategy can be treated as a special case of SFCC in which the original data space is the feature space.

Feature engineering techniques involved in this study include distance-based methods (Euclidean and cosine), correlation-based methods (Pearson [30] and Spearman [31] correlations), and a neural network-based method (autoencoder) [32]. For distance and correlation based methods, the distance/correlation matrix for cells subject to clustering is directly used as their features, and the distance/correlation matrix between cells subject to classification and clustering were used to construct features for cells undergoing classification. For autoencoder, the gene expression data of cells for clustering are used to train a neural network model first and then all cells are projected into a feature space through the encoding function of the trained model. To obtain evaluation results independent of clustering algorithms, we use silhouette values [33] to examine the global performance of these feature engineering methods. Upon the global evaluation, we then select the most effective method, SSCC, the SFCC with Spearman correlation as the feature construction method, to do further evaluations.

### scRNA-seq datasets used in this study

We used seven scRNA-seq datasets to evaluate the clustering performance in feature space. These include the Kolodziejczyk dataset [34], Pollen dataset [8], Usoskin dataset [9], Zeisel dataset [10], Zheng dataset [5], PBMC 68 k dataset [18], and Macosko dataset [19]. Detailed descriptions of these datasets are listed below.

The Kolodziejczyk dataset [34] contains 704 cells with three clusters, which were obtained from mouse embryonic stem cells under different culture conditions. About 10,000 genes were profiled with high sequencing depth (average 9,000,000 reads per cell, >80% of reads mapped to the *Mus musculus* genome GRCm38 with >60% to exons) using the Fluidigm C1 system and applying the SMARTer Kit to obtain cDNA and the Nextera XT Kit for Illumina library preparation.

The Pollen dataset [8] contains 249 cells with 11 clusters, which were obtained from skin cells, pluripotent stem cells, blood cells, neural cells, *etc*. Either low or high sequencing depth based on the C1 Single-Cell Auto Prep Integrated Fluidic Circuit, the SMARTer Ultra Low RNA Kit, and the Nextera XT DNA Sample Preparation Kit was used to depict the gene expression profiles of individual cells (∼50,000 reads per cell).

The Usoskin dataset [9] contains 622 mouse neuronal cells with four clusters, *i.e.*, peptidergic nociceptor-containing, nonpeptidergic nociceptor-containing, neurofilament-containing, and tyrosine hydroxylase-containing cells. The neuronal cells were picked with a robotic cell-picking setup and positioned in wells of 96-well plates before RNA-seq (1,140,000 reads and 3574 genes per cell).

The Zeisel dataset [10] contains 3005 cells from the mouse brain with nine major subtypes. The gene expression levels were estimated by counting the number of unique molecular identifiers (UMIs) obtained by Drop-seq.

The Zheng dataset [5] contains 5063 T cells from five patients with hepatocellular carcinoma. Nine subtypes of samples were prepared according to the tissue types and cell types, and then subject to Smart-seq2 for gene expression profiling (∼1,290,000 uniquely mapped read pairs per cell).

The PBMC 68 k dataset [18] contains 68,578 peripheral blood mononuclear cells (PBMCs) of a healthy human subject. This cell population includes eleven major immune cell types. Gene expression was profiled using the 10× Genomics GemCode platform, and 3′UMI counts were used to quantify gene expression levels with their customized computational pipeline.

The Macoskco dataset [19] contains 49,300 mouse retina cells without known distinct clusters. The gene expression levels were estimated by counting the number of UMIs obtained by Drop-seq. Cells were further clustered into 39 subtypes by the authors based on the Seurat algorithm.

### Data preprocessing

The first four datasets (*i.e.*, the Kolodziejczyk, Pollen, Usoskin, and Zeisel datasets) have been widely used for evaluating clustering algorithms, of which the preprocessed data have been included in the SIMLR software package for test use (https://github.com/BatzoglouLabSU/SIMLR). We downloaded these four datasets from the Matlab subdirectory of the SIMLR package, and then selected the top 5000 most informative genes (with both the average and the standard deviation of log_2_-transformed expression values >1) for subsequent analysis. If the number of genes in a dataset was smaller than 5000, then all the genes in the dataset were retained for further analysis. For the Zheng dataset, one patient (P0508) was selected for comparison of different clustering algorithms, which had 1020 T cells with eight subtypes defined by the tissue sources and the cell surface markers. Genes with both the average and the standard deviation of log_2_-transformed expression values >1 were retained and then the transcripts per million (TPM) values were used for clustering evaluation. For the PBMC 68 k dataset, the preprocessing pipeline described in the original report [18] was used to prepare data for clustering (https://github.com/10XGenomics/single-cell-3prime-paper). For the Macoskco dataset, the UMI counts were used for evaluation without gene filter.

### Consistency between true labels and the original as well as the projected data

**Figure 2:**
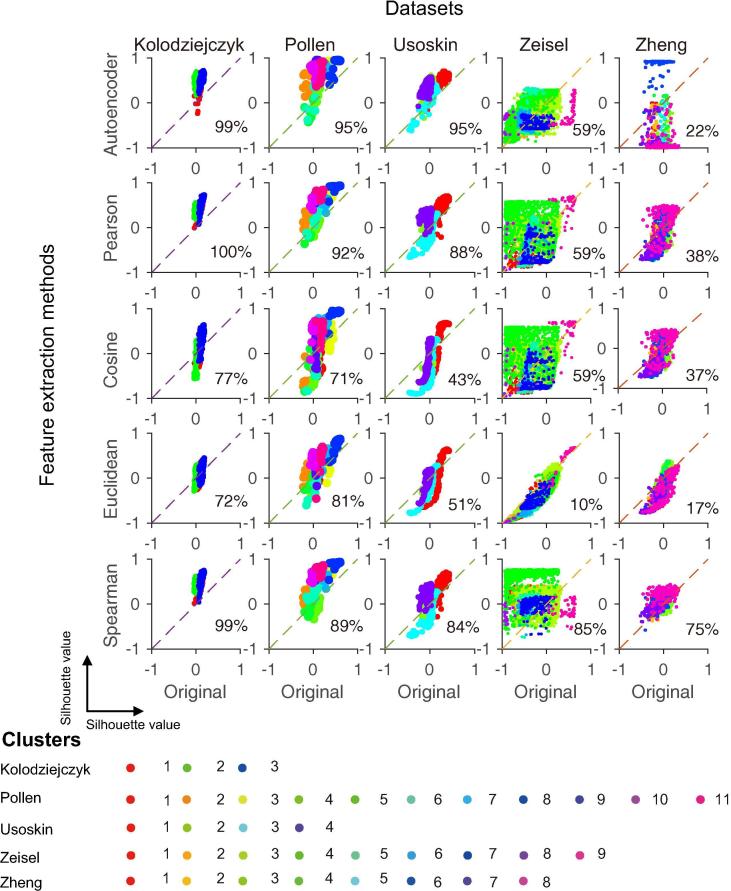
**Consistency with true cluster labels between engineered features and the original data of five datasets** In each plot, each dot represents a cell. Silhouette values calculated using true cluster labels and the original data are shown on X axis, whereas silhouette values calculated using true cluster labels and the engineered features are shown on Y axis. Silhouette value at 1 represents perfect match between labels and features, whereas silhouette value at −1 indicates that the cell might be mis-clustered. The percentage in the plotting area of each plot indicates the fraction of cells above the diagonals. The five datasets tested are the Kolodziejczyk dataset [34], Pollen dataset [8], Usoskin dataset [9], Zeisel dataset [10], and Zheng dataset [5].

The silhouette value [33] is used to measure the consistency between the true labels and the original as well as the projected data. Given a dataset with n samples and a clustering scheme, a silhouette value is calculated for each sample. For a sample i, its silhouette value $s_{i}$ is calculated according to the following formula:

(1) $$s_{i}=\frac{b_{i}-a_{i}}{\text{max}\{a_{i},b_{i}\}}$$

where $a_{i}$ is the average dissimilarity of sample i to samples in its own cluster and$b_{i}$ is the lowest average dissimilarity of sample i to any other cluster of which sample i is not a member. The values of $s_{i}$ range from −1 to 1. A value close to 1 means that sample i is well matched to its cluster, whereas a value close to −1 means that sample i would be more appropriate if it is classified into its neighboring cluster. For each feature construction method, the median silhouette value of all the cells after projection was used to evaluate its consistency with the true cluster labels. The fraction of cells that have silhouette values increased after projection compared to the original data (*i.e.*, the fraction of cells above the diagonal in Figure 2) was also used to evaluate the feature construction methods.

### Clustering accuracy/consistency evaluation

Normalized mutual information (NMI) [35] was used to evaluate the accuracy of various clustering results. Given two clustering schemes $A=\{A_{1},\cdots ,A_{R}\}$ and $B=\{B_{1},\cdots ,B_{s}\}$, the overlap between A and B can be represented through the contingency table C (also named as confusion matrix) of size R×S, where $C_{\mathrm{ij}}$ denotes the number of cells that are shared by clusters $A_{i}$ and $B_{j}$. Then the normalized mutual information $\mathrm{NM}I_{\left(A,B\right)}$ of the two clustering schemes A and B is defined as follows.

(2) $$\mathrm{NM}I_{\left(A,B\right)}=\frac{-2\sum_{i=1}^{R}\sum_{j=1}^{S}C_{\mathrm{ij}}\log\left(\frac{C_{\mathrm{ij}}\times n}{C_{i-}\times C_{-j}}\right)}{\sum_{i=1}^{R}C_{i-}\text{log}\left(\frac{C_{i-}}{n}\right)+\sum_{j=1}^{S}C_{-j}\text{log}\left(\frac{C_{-j}}{n}\right)}$$

where *n* is the number of total cells, $C_{i-}$ is the number of cells assigned to cluster i in the clustering scheme A and $C_{-j}$ is the number of cells assigned to cluster j in the clustering scheme B. If A is identical to B, $\mathrm{NM}I_{\left(A,B\right)}=1$. If A and B are completely different, $\mathrm{NM}I_{\left(A,B\right)}=0$. When true cluster labels were available, the NMI values between true cluster labels and various clustering results were used to evaluate the clustering accuracy. When true cluster labels were not available, NMI was used to evaluate clustering consistency between different subsampling rates in this study. Besides NMI, we also used Rand index and adjusted Rand index to evaluate clustering accuracy and consistency, and obtained similar observations.

### Clustering and classification algorithms

Many clustering algorithms are available. We selected five widelyused clustering algorithms in this study to evaluate the impacts of Spearman correlation-based feature construction method. These five algorithms include three general clustering algorithms which were designed initially not for scRNA-seq data, *i.e.*, affinity propagation (AP) [36], *k*-means [23], and *k*-medoids [37], and two algorithms that were specially designed for clustering of scRNA-seq data, *i.e.*, SC3 [12] and SIMLR [13]. *k*-means and *k*-medoids are pure clustering algorithms that partition samples into groups while AP, SC3, and SIMLR inherently include feature construction techniques. All these clustering algorithms were evaluated on five small-scale datasets (the Kolodziejczyk, Pollen, Usoskin, Zeisel, and Zheng datasets), while only SC3 was evaluated on the PBMC 68 k dataset and only *k*-means was evaluated on the Macoskco dataset for simplicity. Parameters (ks = 10:12, gene_filter = FALSE, biology = FALSE, svm_max = 5000) were used for SC3 (default), whereas parameters (ks = 11, gene_filter = FALSE, biology = FALSE, svm_max = 200) were used for SC3 + SSCC. On the Macoskco dataset, ∼5% and 10% cells were randomly picked out for clustering analyses. We used the *k*-nearest neighbor algorithm for classifying unsubsampled cells, which is robust to parameter selection.

## Results

### Feature construction can greatly improve the consistency of cell features and the reference cell labels

First we evaluated whether feature extraction methods can improve clustering results of scRNA-seq data. We calculated the silhouette values to evaluate the consistency between cell features extracted using various methods and the reference labels. Silhouette values are frequently used to indicate whether a sample is properly clustered. But here we can use silhouette values to reversely indicate whether the extracted features are properly consistent with the reference cell labels. By comparing with silhouette values of the original scRNA-seq data, we observed that most of the evaluated feature-extracting methods can improve the silhouette values for many cells in multiple datasets (Figure 2). For the Kolodziejczyk [34] and Pollen [8] datasets, all the five feature-extraction methods improved the silhouette values compared with the original data. For the Usoskin [9] dataset, all methods showed significantly better performance except Euclidean and cosine. For the Zeisel [10] dataset, only Spearman correlation resulted in improvement for >80% cells compared with the original data, while other feature extraction methods except Euclidean resulted in little improvement. Euclidean resulted in even worse results for the Zeisel dataset, indicating low robustness. For the Zheng [5] dataset, most methods failed except the Spearman correlation method. The Spearman correlation-based feature extraction method consistently improved the accordance between cell features and labels on all the five datasets. Considering the robustness of Spearman’s correlation-based method and the great improvement of silhouette values of single cells, we evaluated the accuracy, robustness, and efficacy of SSCC in the next section.

### Clustering accuracy of the total cells is enhanced in feature space when subsampling is applied

**Figure 3:**
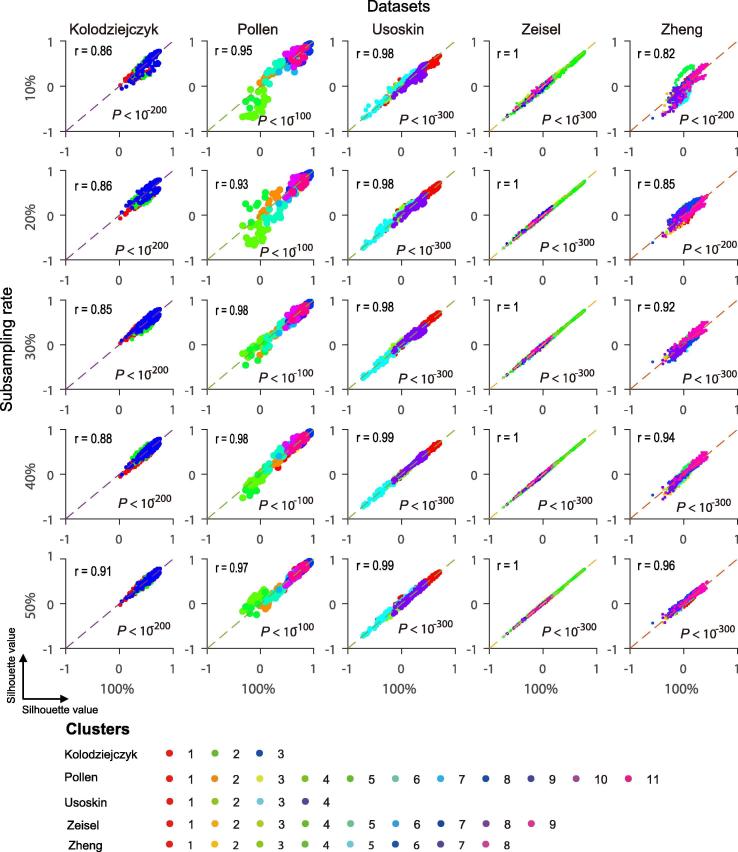
**Silhouette values between Spearmancorrelation features and true cluster labels are independent of subsampling rates in five datasets** Spearman correlation features were constructed at various subsampling rates of the original data in the five datasets. In each plot, each dot represents a cell. Silhouette values of Spearman correlation features constructed with 100% cells are shown on X axis, whereas silhouette values of Spearman correlation features constructed with 10%, 20%, 30%, 40%, and 50% cells in each dataset are shown on Y axis. Pearson correlation between X and Y axes was calculated, where the correlation coefficient (*r*) is provided in the upper triangle and the corresponding *P* value is provided in the lower triangle of each plot.

While subsampling can greatly boost the efficiency of clustering of large scRNA-seq data, it often compromises the clustering accuracy. We observed that the improvements of silhouette scores by SSCC were robust to subsampling fluctuations (Figure 3). For all the five datasets evaluated, the silhouette values of Spearman correlation-based features were almost unchanged with subsampling rates (Figure 3). These data suggest that features constructed using SSCC at low subsampling rates may contain information approximate to that with total cell populations.

**Figure 4:**
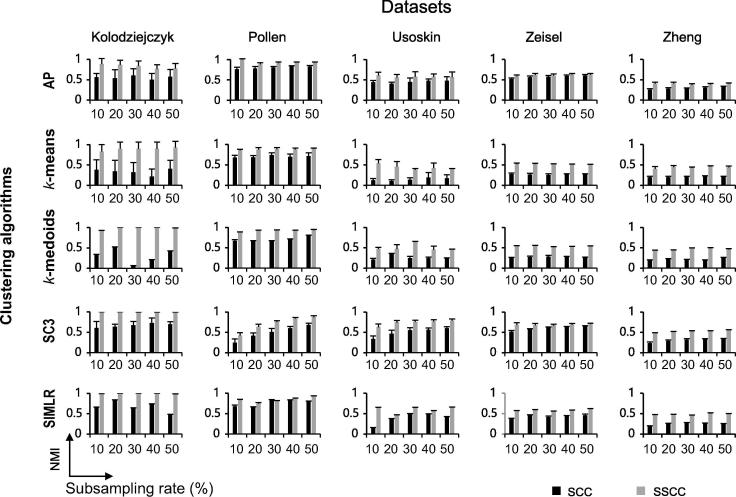
**Clustering performance comparison between SCC and SSCC with varied subsampling rates in five datasets** Clustering accuracy using SCC and SSCC was measured at various subsampling rates of the original data in the five datasets, *i.e.*, the percentage of cells used in clustering. The clustering accuracy is indicated using NMI. For each subsampling rate, calculations were repeated for ten times, based on which the average and the standard deviation of the clustering accuracy were calculated and plotted. NMI, normalized mutual information; SSCC, Spearman subsampling-clustering-classification; AP, affinity propagation.

We further evaluated whether the improved silhouette values can be translated into clustering accuracy. By evaluating five clustering algorithms including *k*-means, *k*-medoids, AP, SC3, and SIMLR, we observed that compared to SCC, SSCC can significantly improve the clustering accuracy in terms of NMI, for all the five clustering algorithms on all the benchmark datasets tested (Figure 4). The accuracy improvements measured by ΔNMI range from 0.12 to 0.60 for the Kolodziejczyk dataset, 0.04 to 0.19 for the Pollen dataset, 0.14 to 0.37 for the Usoskin dataset, 0.02 to 0.28 for the Zeisel dataset, and 0.10 to 0.28 for the Zheng dataset, depending on the algorithms and subsampling rates chosen. Other accuracy metrics including Rand index, adjusted Rand index, and adjusted mutual information reveal the same trends (data not shown), suggesting that SSCC can greatly enhance the power of multiple clustering algorithms when subsampling is used.

### Clustering consistency between different subsampling runs is also greatly improved with SSCC

**Figure 5:**
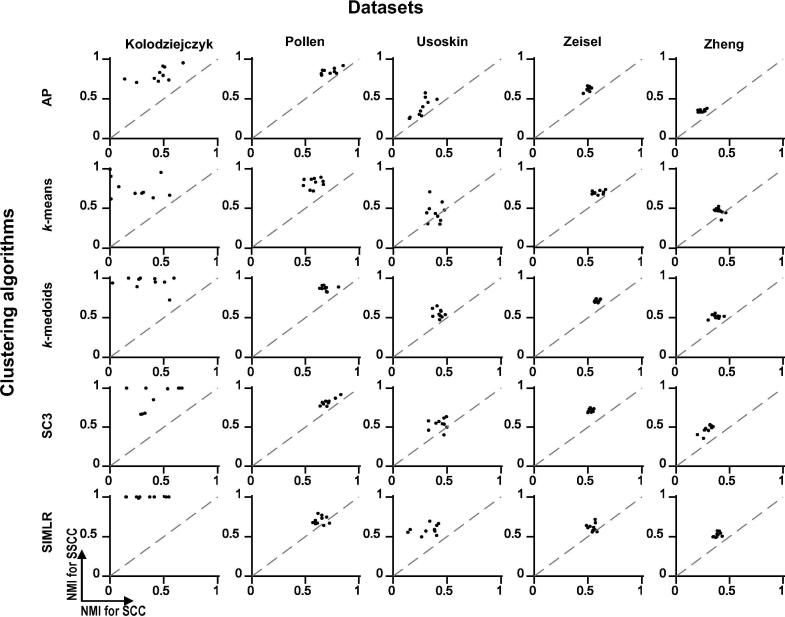
**Comparison of clustering consistency between SSCC and SCC for five datasets** The consistency (measured by NMI) of clustering between using 10% cells and that using 50% cells with SCC is shown on X axis, whereas consistency (measured by NMI) of clustering between using 10% cells and that using 50% cells with SSCC is shown on Y axis. Subsamplings were repeated for ten times and each subsampling result was processed using five clustering algorithms shown on the left.

In practice, the reference cell labels are generally unknown. The confidence of clustering results is often evaluated by the consistency between different algorithms. Due to the subsampling fluctuations, clustering results based on SCC are inconsistent among different subsampling operations. However, in the new framework of SSCC, the consistency was much improved for all evaluated clustering algorithms on all datasets (Figure 5). For the Kolodziejczyk dataset, all the five clustering algorithms had consistency >0.5 (measured by NMI) in SSCC while the corresponding consistency in SCC was much smaller. For the Pollen dataset, SSCC still showed better performance than SCC although both frameworks had high clustering consistency. Similar trends were observed on the Usoskin, Zeisel, and Zheng datasets.

### Application of SSCC to large scRNA-seq datasets with or without reference cell labels

Besides the aforementioned five scRNA-seq datasets, we further tested SSCC on two additional large scRNA-seq datasets. One is the PBMC 68 k dataset [18], which contains 10× Genomics-based expression data for 68,578 blood cells from a healthy donor. The other is the Macoskco dataset [19], which contains 49,300 mouse retina cells lacking of experimentally determined cell labels. The large cell numbers generally prohibit classic scRNA-seq clustering algorithms running on a desktop computer, thus providing two realistic examples to demonstrate the performance of SCC and SSCC.

**Figure 6:**
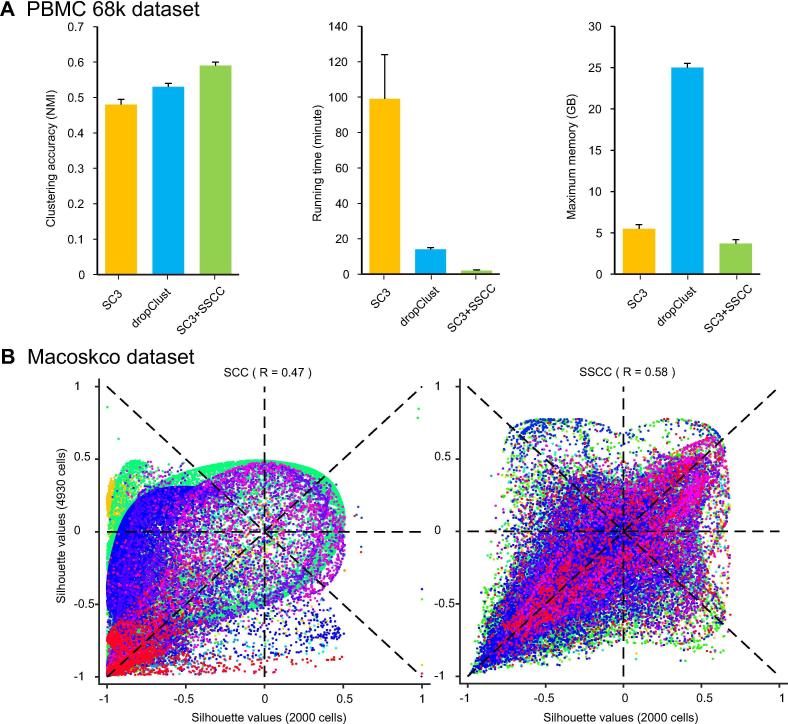
**Clustering performance evaluation of SSCC on two extremely large scRNA-seq datasets** **A.** Performance comparison between SC3 (default), dropClust, and SC3 + SSCC on the PBMC 68 k dataset [18] in terms of clustering accuracy, running time and maximum memory required. In total 5000 cells were subsampled for SC3 (default), while 200 cells were subsampled for SC3 + SSCC. **B.** Consistency comparison between SSCC (on the right) and SCC (on the left) evaluated on 49,300 mouse retina cells in the Macosko dataset [19]. Silhouette values of two clustering schemes (using 2000 cells and 4930 cells, separately) were plotted and then Pearson correlation coefficients were calculated. The 39 cell clusters were colored according to cluster labels based on ∼10% cells and original expression data.

For the PBMC 68 k dataset, we compared SSCC with SCC using SC3 [12] as the clustering algorithm. The SC3 software package inherently applies an SCC strategy to handle large scRNA-seq datasets. By default, if a dataset has more than 5000 cells, the SCC strategy will be triggered, with 5000 cells randomly subsampled for SC3 clustering and the other cells for classification by SVM. We applied SC3 to the PBMC 68 k dataset on a desktop computer with 8 GB memory and 3 GHz 4-core CPU and repeated ten times. The average clustering accuracy of SC3 in terms of NMI was 0.48, the calculation took 99 min on average, and the maximum memory usage exceeded 5.6 GB (Figure 6A). With the SSCC strategy, the average clustering accuracy reached 0.59, representing ∼21% increase over SC3 with the default parameters. It is of note that the computation time was dramatically reduced to 2.2 min on average, representing a 50-fold acceleration. Meanwhile, the maximum memory usage of SC3 + SSCC was 3.7 GB, saving >33% compared to that of SC3 with the default parameters. Compared to dropClust [20], a clustering algorithm specialized for large scRNA-seq datasets, SC3 + SSCC also demonstrated superior performance in terms of clustering accuracy, speed, and memory usage (Figure 6A).

For the Macoskco dataset, using *k*-means as the clustering algorithm and *k*-nearest neighbors for classification, the SCC strategy resulted in great average silhouette difference (0.29) between two subsampling schemes (−0.80 with 5% cells and −0.51 with 10% cells), whereas the difference using SSCC became negligible (0.01). The NMI values between the two subsampling schemes were 0.60 and 0.69 when using SCC and SSCC, respectively. Pearson correlation coefficients of silhouette values between the two subsampling schemes were increased from 0.47 to 0.58 when switching from SCC to SSCC (Figure 6B).

All these metrics demonstrate that SSCC can not only greatly improve the clustering efficiency and accuracy for large-scale scRNA-seq datasets, but also can greatly improve the consistency.

## Discussion

The availability of large-scale scRNA-seq data raises urgent need for efficient and accurate clustering tools. Currently a few scRNA-seq data analysis packages have been proposed to address this challenge. Of these tools, SC3 [12], Seurat [11], and dropClust [20] adopt a SCC strategy, bigScale [21] employs a convolution strategy to merge similar single cells into mega cells by a greedy-searching algorithm, and SCANPY [22] used Python as the programming language to accelerate the clustering process. Although these strategies greatly boost the efficiency of large scRNA-seq data analysis, there exists much room for further improvement. Particularly the SCC strategy suffers from biases introduced by subsampling which may greatly decrease the clustering accuracy and robustness, although it can reduce the computational complexity from O(n^2^) to O(n). Here we introduce feature engineering and projecting techniques into the SCC framework and propose SFCC as an alternative. Specially, with Spearman correlations as the feature engineering and projecting methods, we formulate a framework named as SSCC, which can significantly improve clustering accuracy and consistency for many general and speciallydesigned clustering algorithms. Evaluations on real scRNA-seq datasets, which cover a wide range of scRNA-seq technologies, sequencing depths, and organisms, demonstrate the robustness of the superior performance of SSCC. Therefore, SSCC is expected to be a useful computational framework that can further unleash the great power of scRNA-seq in the future.

## Authors’ contributions

XR and ZZ designed the study. XR and LZ collected the data, implemented the software, and did the analysis. XR and ZZ wrote the manuscript. All authors read and approved the final manuscript.

## Competing interests

The authors have declared no competing interests.
